# Reprogramming cancer immunity with next-generation combination therapies

**DOI:** 10.3389/fcell.2025.1652047

**Published:** 2025-08-28

**Authors:** Nikolaos C. Kyriakidis, Carolina E. Echeverría, Jhommara Bautista, Sebastián Rivera-Orellana, María José Ramos-Medina, Camila Salazar-Santoliva, Juan S. Izquierdo-Condoy, Esteban Ortiz-Prado, Santiago Guerrero, Andrés López-Cortés

**Affiliations:** ^1^ Center for Hematology and Regenerative Medicine, Department of Medicine Huddinge, Karolinska Institute, Stockholm, Sweden; ^2^ Department of Medicine, New York University Grossman School of Medicine, New York, NY, United States; ^3^ Cancer Research Group (CRG), Faculty of Medicine, Universidad de Las Américas, Quito, Ecuador; ^4^ Facultad de Ciencias, Campus de A Zapateira, Universidade da Coruña, A Coruña, Spain; ^5^ German Cancer Research Center (DKFZ), Faculty of Biosciences, Heidelberg University, Heidelberg, Germany; ^6^ One Health Research Group, Faculty of Health Science, Universidad de Las Américas, Quito, Ecuador; ^7^ Laboratorio de Ciencia de Datos Biomédicos, Facultad de Ciencias Médicas de la Salud y de la Vida, Universidad Internacional del Ecuador, Quito, Ecuador

**Keywords:** cancer immunotherapy, immune checkpoints, adoptive cell therapy, next-generation therapeutics, biomarker-guided precision medicine, tumor microenvironment

## Abstract

Cancer immunotherapy has fundamentally reshaped oncology by harnessing the immune system to eliminate malignant cells. Immune checkpoint inhibitors targeting CTLA-4 and PD-1/PD-L1 have achieved durable remissions in select cancers, yet most patients exhibit resistance due to tumor heterogeneity, immunometabolic rewiring, and the immunosuppressive tumor microenvironment. To address these limitations, next-generation immunotherapies have emerged, targeting multiple layers of immune regulation. These include co-inhibitory and co-stimulatory checkpoint modulators, bispecific antibodies, adoptive cell therapies, cancer vaccines, oncolytic viruses, cytokine-based strategies, and synthetic immunomodulators that activate innate sensors. Nanotechnology and *in vivo* immune engineering further enhance specificity, reduce toxicity, and broaden applicability. Combination immunotherapy has become central to overcoming resistance, with rational regimens integrating ICIs, cytokines, vaccines, and targeted agents. Biomarker-guided strategies, leveraging tumor mutational burden, immune cell infiltration, and multi-omic profiling, are enabling personalized approaches. However, immune-related adverse events and variability in therapeutic responses necessitate predictive biomarkers and improved patient stratification. Emerging frontiers include microbiome-targeted interventions, chronotherapy, and AI-driven modeling of tumor–immune dynamics. Equally critical is ensuring global equity through inclusive trial design, diverse biomarker validation, and expanded access to cutting-edge therapies. This review provides a comprehensive analysis of multimodal immunotherapeutic strategies, their mechanistic basis, and clinical integration. By unifying innovation in immunology, synthetic biology, and systems medicine, next-generation cancer immunotherapy is poised to transition from a transformative intervention to a curative paradigm across malignancies.

## Introduction

Cancer immunotherapy has transformed the therapeutic landscape of oncology by harnessing the immune system to detect and eliminate malignant cells^1^. Immune checkpoint inhibitors (ICIs), particularly those targeting cytotoxic T lymphocyte-associated antigen 4 (CTLA-4) and programmed death 1 (PD-1)/programmed death-ligand 1 (PD-L1), have achieved durable clinical responses across multiple malignancies, including melanoma, non-small cell lung cancer (NSCLC), and renal cell carcinoma (RCC) (Nagasaki et al., 2022; Sun et al., 2023; Pardoll, 2012). However, despite these advances, a substantial proportion of patients do not respond or eventually relapse. Mechanisms of resistance include intratumoral heterogeneity, immunometabolic rewiring such as the Warburg effect, and the immunosuppressive architecture of the tumor microenvironment (TME) (Schoenfeld and Hellmann, 2020; Usset et al., 2024; Echeverría et al., 2024; Ribas and Wolchok, 2018).

These limitations have catalyzed efforts to expand the immunotherapeutic arsenal beyond classical ICIs, leading to the development of novel modalities that engage diverse components of the immune system. These include cancer vaccines that deliver tumor-specific antigens to dendritic cells (DCs), oncolytic viruses (OVs) that promote direct tumor lysis and immune activation (Shalhout et al., 2023), bispecific antibodies (BsAbs) that redirect T cells to tumor cells (Trabolsi et al., 2024; Herrera et al., 2024), adoptive cell therapies (ACTs), such as chimeric antigen receptor (CAR) and T cell receptor (TCR)-engineered cells, confer engineered cytotoxicity (Peng et al., 2024; Kapetanovic et al., 2024), and cytokine-based interventions (Hafler, 2007). Collectively, these approaches target multiple layers of immunological control, from enhancing antigen presentation and T cell priming to reprogramming the TME, to achieve robust and durable antitumor immunity (Morad et al., 2021; Dagher et al., 2023; Goswami et al., 2024). Additionally, synthetic immunomodulators, such as engineered cationic helical polypeptides that activate innate immune sensors (Lee et al., 2024), and microbiome-based interventions that reshape systemic and local immunity (Zheng et al., 2020), are expanding the therapeutic landscape. Nanotechnology-driven delivery systems further enable precise antigen targeting, reduce systemic toxicity, and support *in vivo* immune cell engineering.

Combination immunotherapy has emerged as a cornerstone of modern clinical development (Goswami et al., 2024). Rationally designed regimens, such as dual ICI blockade (anti–PD-1 plus anti–CTLA-4), checkpoint inhibition combined with co-stimulatory agonists (GITR, OX40, CD40), and combinations with radiotherapy, chemotherapy, or targeted agents, are actively being explored to address immune escape and resistance (Zhang et al., 2025; Dai et al., 2024; Krishnamoorthy et al., 2021). Increasingly, biomarker-guided selection and molecular profiling are guiding the deployment of these combinations, enabling personalized and context-specific strategies. This review provides a comprehensive analysis of emerging immunotherapeutic strategies and their mechanistic underpinnings, clinical advances, and potential for integration into rational combination regimens. Particular emphasis is placed on how these multimodal interventions converge to rewire cancer immunity, overcome therapeutic resistance, and extend the promise of durable clinical benefit across diverse tumor types.

## Cancer vaccines

### Preventive cancer vaccines

Preventive vaccines are designed to reduce the risk of cancer by targeting oncogenic viral infections (Bautista and Lopez-Cortes, 2025). Currently, two vaccines are approved for cancer prevention: the human papillomavirus (HPV) vaccine and the hepatitis B virus (HBV) vaccine. Prophylactic HPV vaccines, available in bivalent and quadrivalent formulations, have demonstrated over 90% efficacy in preventing cervical cancer and other HPV-related malignancies (Brisson et al., 2020). These vaccines also confer indirect protection to men through herd immunity (Drolet et al., 2019). Similarly, HBV vaccination is a cornerstone of public health strategies to prevent HBV-induced HCC. Integration into national immunization programs has significantly decreased HBV incidence and reduced acute infections, particularly among children and adolescents (Chae and Tak, 2023). Vaccination at birth prevents chronic HBV infection in over 90% of cases. However, challenges persist in preventing vertical transmission, especially from hepatitis B e-antigen (HBeAg)-positive mothers (Chen et al., 2017). Advances such as Heplisav-B offer improved immunogenicity and safety over older vaccines like Engerix-B, providing enhanced protection for non-responders (Splawn et al., 2018). In addition, efforts are underway to develop therapeutic vaccines targeting chronic HBV by enhancing immune clearance where prophylactic vaccination is insufficient (Hudu et al., 2024).

### Therapeutic cancer vaccines

Therapeutic vaccines are designed to induce an immune response against established tumors. These vaccines deliver tumor-specific or tumor-associated antigens (TAAs), to APCs, particularly DCs, which in turn activate CTLs to recognize and kill cancer cells (Saxena et al., 2021). The goals of therapeutic vaccines include halting tumor progression, eradicating residual disease, and preventing relapse. Technological advancements in whole-exome/genome sequencing and bioinformatics have enabled the identification of neoantigens—tumor-specific mutated proteins with high immunogenicity and low tolerance, enabling personalized vaccine design (Brandenburg et al., 2024). In addition to neoantigens, shared TAAs expressed across multiple tumor types are commonly targeted (Saxena et al., 2021). Therapeutic vaccines function by enhancing antigen presentation, stimulating DC maturation, and promoting durable T cell–mediated immune responses within the TME, where both innate (natural killer (NK) cells, macrophages, neutrophils) and adaptive (T and B cells) immune cells coordinate antitumor immunity (Fan et al., 2023).

**FIGURE 1:**
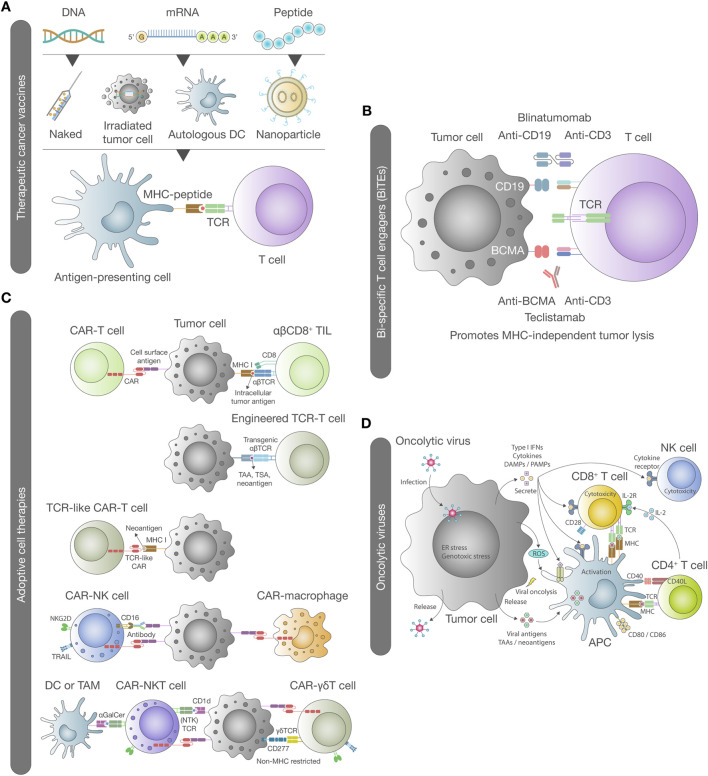
Landscape of cancer immunotherapies. **(A)** Therapeutic cancer vaccines stimulate endogenous T cell immune responses against tumor antigens. This process begins with the uptake, processing, and presentation of antigens by dendritic cells. Immune activation can be induced through various methods, including the injection of naked or nanoparticle-encapsulated DNA, RNA, or peptides, *ex vivo* pulsing of autologous dendritic cells, and the administration of irradiated tumor cells. Antigen presentation is mediated by specific human leukocyte antigens (HLA) within the major histocompatibility complex (MHC) I and II, which activate CD8^+^ and CD4^+^ T cells, respectively. **(B)** BiTEs have emerged as a key class of immunotherapeutic agents in oncology. These recombinant proteins consist of two antigen-binding modules connected by a short linker sequence or a shared Fc domain. One module binds to a tumor-associated antigen (TAA), while the other engages a T cell activation molecule, such as CD3 on the T cell receptor (TCR) complex. The simultaneous binding of BiTEs to both the tumor antigen and the TCR leads to T cell activation, the formation of immune synapses, and tumor cell lysis. BiTEs can be engineered to target various TAAs, offering broad therapeutic potential across multiple cancer types. **(C)** Adoptive cell therapies involve the *ex vivo* expansion and infusion of autologous or allogeneic immune cells to enhance tumor eradication. The most widely used adoptive cell therapies include tumor-infiltrating lymphocytes (TILs) and genetically modified T cells expressing transgenic TCRs or chimeric antigen receptors (CARs). Unlike TILs and transgenic TCR T cells, CAR-T cells function independently of MHC molecules and can be designed to target a wide range of TAAs. Additionally, gene modification strategies have been developed to enhance adoptive cell therapies efficacy in solid tumors. These include engineering natural killer (NK) cells and macrophages to express transgenic TCRs, NK cell receptors, CARs, or TCR-like CARs. **(D)** Oncolytic viruses represent a dual-action cancer therapy that combines direct cancer cell lysis with immune system activation. Upon infection, cancer cells initiate an antiviral response involving endoplasmic reticulum and genotoxic stress, leading to increased reactive oxygen species (ROS) and the production of antiviral cytokines, particularly type I interferons (IFNs). These signals activate immune cells, including antigen-presenting cells, CD8^+^ T cells, and NK cells. Oncolysis releases viral progeny, pathogen-associated molecular patterns (PAMPs), danger-associated molecular patterns (DAMPs), and TAAs, including neoantigens. The released viral progeny propagates the infection, while PAMPs and DAMPs stimulate immune receptors such as Toll-like receptors (TLRs). This immune-stimulatory environment enhances antigen presentation and promotes the generation of immune responses against both virally infected and uninfected cancer cells expressing TAAs and neoantigens.

To achieve these goals, various vaccine platforms have been developed. DNA, RNA, and peptide-based vaccines deliver tumor antigens in forms that are processed and presented by APCs, inducing CTL responses (Kyriakidis et al., 2021). Nanoparticle-based systems further enhance vaccine efficacy by stabilizing antigens, improving cellular uptake, and directing delivery to specific immune cell subsets (Brandenburg et al., 2024). *Ex vivo*–loaded DC vaccines involve isolating DCs from the patient, pulsing them with tumor antigens *in vitro*, and reinfusing them to generate a robust and targeted immune response (Chiang et al., 2013). Irradiated tumor cell vaccines use tumor cells that have been rendered non-replicative yet maintain antigenicity, often engineered to secrete immune-activating factors that boost T cell priming (Curry et al., 2016). A notable example is Sipuleucel-T, approved in 2010 for metastatic castration-resistant prostate cancer. This autologous DC-based vaccine loads patient-derived DCs with a prostate antigen (PAP-GM-CSF fusion protein) and reinfuses them to stimulate a targeted immune response (Kantoff et al., 2010) (Figure 1A).

Beyond conventional vaccines, innovative immunotherapeutic strategies continue to emerge. One of the most established is intravesical *Bacillus* Calmette–Guérin (BCG) therapy for early-stage non-muscle invasive bladder cancer (NMIBC). BCG elicits a strong, localized immune response that helps prevent tumor recurrence. For patients who fail BCG therapy, the FDA-approved gene therapy nadofaragene firadenovec (Adstiladrin) provides a new option. This therapy uses a replication-deficient adenoviral vector to deliver human interferon alfa-2b cDNA directly to the bladder, showing high complete response rates in Phase III trials (Konety et al., 2024; Narayan et al., 2024).

Ongoing clinical trials continue to expand the therapeutic landscape. At Memorial Sloan Kettering Cancer Center, an mRNA-based vaccine targeting pancreatic cancer has shown promise in Phase I trials. This personalized vaccine induced robust CD8^+^ T cell responses that persisted for up to 4 years and correlated with reduced relapse at a 3-year follow-up (Sethna et al., 2025). Overall, vaccine development for cancer treatment is one of the most developing fields currently. Future investigations and ongoing clinical trials will further delineate the optimal targets of the immune response, dosage and vaccine platforms, including their integration into combination therapies aimed at maximizing patient outcomes in the face of a heterogeneous disease landscape.

## Bi-specific T cell engagers and dual-affinity retargeting antibodies

In recent decades, BsAbs have emerged as a promising therapeutic strategy for hematologic malignancies (Tian et al., 2021) and are increasingly being explored for the treatment of solid tumors, including lung, breast, pancreatic, and prostate cancers (Palecki et al., 2024; Simão et al., 2023). Among the more than 100 bispecific formats developed, bi-specific T cell engagers (BiTEs) stand out due to their design and ongoing structural innovations (Kühl et al., 2022). BiTEs are recombinant proteins composed of two antigen-binding modules connected by a short linker or a shared Fc domain. One module binds a TAA on cancer cells, while the other engages the CD3-TCR complex on T cells. This dual binding facilitates the selective recruitment and activation of T cells against tumor cells, leading to enhanced cytotoxic responses and effectively harnessing the body’s immune system to combat cancer. The modulation of BiTEs allows them to be engineered against virtually any cell surface TTA, providing a versatile platform for the treatment of diverse cancer types (Simão et al., 2023). As of 2025, at least nine FDA-approved BiTEs have been approved, including BiTEs and BiTE-like constructs. Blinatumomab, targeting CD19 on B cells, is approved for the treatment of relapsed or refractory (r/r) B cell precursor acute lymphoblastic leukemia (BCP-ALL) and BCP-ALL with minimal residual disease (Goebeler and Bargou, 2016; Sun et al., 2015). Teclistamab, a bispecific mAb targeting B cell maturation antigen (BCMA), is indicated for r/r multiple myeloma (Guo et al., 2024). Similarly, Talquetamab targets GPRC5D on myeloma cells and has been approved for r/r multiple myeloma (Keam, 2023). Elranatamab, another BCMA-targeting BiTE, is approved for heavily pretreated r/r multiple myeloma (Dhillon, 2023). Tebentafusp, distinct from conventional BiTEs, utilizes a soluble high-affinity TCR to target a gp100-derived peptide presented by MHC class I complexes and is approved for uveal melanoma (Dhillon, 2022). Epcoritamab, which engages both CD20 on B cells and CD3 on T cells, is approved for r/r diffuse large B cell lymphoma (DLBCL) (Frampton, 2023). Tarlatamab, targeting delta-like ligand 3 (DLL3), has been approved for SCLC in patients who have progressed after platinum-based chemotherapy (Dhillon, 2024). Glofitamab, a 2:1 CD20 × CD3 BiTE, is FDA-approved for relapsed/refractory DLBCL after ≥2 prior therapies. Its unique bivalent CD20 binding enhances tumor targeting and T cell activation, with obinutuzumab pre-treatment reducing CRS (Hutchings et al., 2021). Mosunetuzumab, another CD20 × CD3 BiTE, is approved for relapsed/refractory follicular lymphoma. It uses a step-up dosing regimen to mitigate CRS and has shown high complete response rates in heavily pretreated patients (Sehn et al., 2025).

Despite the success of BiTEs in hematologic malignancies, their efficacy in solid tumors remains limited due to barriers such as poor T cell infiltration and the presence of an immunosuppressive TME. To address these challenges, innovative strategies are being developed, including checkpoint inhibitory T cell engagers (CiTEs) (Szijj et al., 2023), simultaneous multiple interaction T cell engagers (SMiTEs) (Correnti et al., 2018), and oncolytic virus-mediated BiTE delivery systems. These novel approaches aim to enhance T cell activation, overcome immune suppression, and improve therapeutic outcomes in solid tumors (Heidbuechel and Engeland, 2021). Additionally, combining BiTEs with other modalities, such as CAR-T cell therapy and ICIs, is being actively explored to further boost anti-tumor responses (Yin et al., 2022). Ongoing clinical studies and trials are essential for refining these therapies, extending patient remission, and minimizing relapse rates (Figure 1B). The expanding repertoire of BsAbs reflects ongoing advances in molecular engineering. Dual-affinity retargeting antibodies (DARTs) have been developed to improve stability and target engagement compared to earlier bispecific designs (Allen et al., 2021). Furthermore, innovations in the engineering of single-chain variable fragments (scFvs), which serve as modular building blocks for bispecific formats, have enabled the simultaneous targeting of multiple tumor antigens (Chen et al., 2016). This strategy is particularly valuable for addressing tumor heterogeneity, a major factor complicating treatment resistance. While the challenges associated with toxicity and solid tumor targeting persist, the precision and adaptability of BiTEs position them as a powerful modality in the evolving landscape of cancer immunotherapy.

### Adoptive cell therapies

ACTs represent a transformative modality in cancer immunotherapy, involving the isolation, expansion, and reinfusion of autologous or allogeneic immune cells to enhance tumor eradication. Among ACT approaches, TILs and genetically engineered T cells are widely employed. The latter includes T cells modified to express transgenic TCRs or CARs. Unlike TILs and TCR-modified T cells, CAR T cells operate independently of MHC-mediated antigen presentation, enabling broader and more versatile targeting of TAAs, a property that has made them a cornerstone in adoptive immunotherapy strategies (Finck et al., 2022) (Figure 1C). CAR T cells have demonstrated remarkable clinical success in hematologic malignancies. To date, six CAR T therapies have received FDA approval: four targeting CD19 (tisagenlecleucel, axicabtagene ciloleucel, lisocabtagene maraleucel, and brexucabtagene autoleucel) and two targeting BCMA (idecabtagene vicleucel and ciltacabtagene autoleucel) for multiple myeloma (Mitra et al., 2023). These therapies show high response rates in B-cell precursor ALL, large B-cell lymphoma (LBCL), mantle cell lymphoma (MCL), and relapsed/refractory multiple myeloma (RMM) (Zugasti et al., 2025; Goyco Vera et al., 2024). Clinical trials have also expanded to target CD22, showing promise in ALL, and further preclinical efforts include dual-targeting strategies to mitigate antigen loss and tumor escape (Abbasi et al., 2023; June et al., 2018).

Structurally, CARs comprise an scFv domain for antigen recognition, a hinge region, a transmembrane domain, and intracellular signaling domains such as CD3ζ and co-stimulatory elements (e.g., CD28 or 4-1BB), which define their generation and impact persistence and function (Safarzadeh Kozani et al., 2022). Second-generation CARs have become the clinical standard, offering enhanced cytotoxicity and memory formation (Hong et al., 2020). New designs now incorporate logic gating, cytokine expression, and safety switches to enhance control and efficacy. Despite success in blood cancers, CAR T cell efficacy in solid tumors remains limited due to several challenges: scarcity of tumor-specific antigens, risk of on-target off-tumor toxicity, antigen heterogeneity, and an immunosuppressive TME that impairs T cell trafficking and function. To overcome these barriers, innovative *in vivo* engineering strategies have emerged, aiming to simplify manufacturing and broaden applicability. One major innovation is *in vivo* CAR T cell generation, which bypasses *ex vivo* cell manipulation and instead employs targeted delivery systems, such as lipid nanoparticles (LNPs), polymeric nanoparticles, viral vectors (e.g., lentivirus, AAV), and bioinstructive scaffolds, to directly engineer immune cells within the body (Li et al., 2025; Hunter et al., 2025). Among these, LNP–mRNA systems have shown particular promise due to their transient expression profiles, lower immunogenicity, and scalable manufacturing, benefiting from platforms used in mRNA vaccines (Kim et al., 2024; Kyriakidis et al., 2021). For instance, maleimide-functionalized LNPs conjugated to CD3-targeting antibodies have successfully delivered CD19 CAR mRNA to splenic T cells in preclinical models, achieving effective B cell depletion with minimal liver toxicity. In addition, Cas9-packaging enveloped delivery vehicles (Cas9-EDVs) allow for simultaneous *in vivo* CAR insertion and gene editing (e.g., TRAC knockout), facilitating allogeneic cell generation while reducing graft-versus-host risks. Virus-mimetic fusogenic nanovesicles (FuNVs) present an alternative by directly inserting preformed CAR proteins into T cells, enabling transient functionality without gene integration (Li et al., 2025; Hunter et al., 2025).

However, the use of CAR T cells is not without risk. The two most significant adverse effects are CRS and immune effector cell-associated neurotoxicity syndrome (ICANS) (Asghar et al., 2023). CRS results from excessive cytokine secretion following T cell activation, presenting with fever, hypotension, and potentially multiorgan dysfunction. ICANS often co-occurs with severe CRS and is characterized by neurologic symptoms such as confusion, seizures, and cerebral edema, likely due to cytokine-induced blood-brain barrier disruption (Santomasso et al., 2023).

As adoptive therapies expand, alternative effector cells like NK cells and macrophages are increasingly being explored for CAR engineering. These innate immune cells offer unique biological properties that may help overcome the limitations faced by CAR T cell therapies, particularly in solid tumors. A leading example of CAR NK development is FT596, the first-in-class, off-the-shelf, induced pluripotent stem-cell (iPSC)-derived CAR NK product. FT596 is genetically engineered to express a CD19-specific CAR, an IL-15 receptor fusion to enhance persistence, and a high-affinity CD16 Fc receptor to facilitate antibody-dependent cellular cytotoxicity (ADCC) (Lin et al., 2023; Bachanova et al., 2021; Rezvani, 2019). In a Phase I trial involving patients with relapsed or refractory B cell malignancies, FT596 demonstrated a favorable safety profile, including an absence of cytokine release syndrome (CRS), and encouraging antitumor activity, underscoring its potential as an allogeneic and low-toxicity platform (Ghobadi et al., 2025).

Parallel efforts have led to the development of CT-0508, a first-in-class autologous CAR macrophage therapy designed to target HER2-expressing solid tumors. This therapy not only mediates direct cytotoxicity through phagocytosis but also remodels the TME by enhancing antigen presentation and promoting T cell infiltration (Klichinsky et al., 2020; Morva et al., 2025). Early-phase clinical evaluation of CT-0508 has demonstrated feasibility, biological activity, and favorable tolerability in patients with advanced HER2-positive cancers (Reiss et al., 2022; Reiss et al., 2025). Complementing these clinical approaches, preclinical models of *in vivo* CAR macrophage engineering, using mannose-targeted LPNs or biodegradable polymeric carriers, have shown promising efficacy in difficult-to-treat tumors such as pancreatic and HCC (Dagher et al., 2023). These examples underscore the growing interest in using innate immune cells to overcome the limitations of CAR T therapy in solid tumors and expand the adoptive cell therapy toolkit.

### Cytokine therapy

Cytokines are small, soluble proteins that mediate cell-to-cell communication and regulate the activation, differentiation, and function of both innate and adaptive immune responses. These key regulators of immune responses have been explored as cancer therapeutics due to their ability to activate and expand immune effector cells (Berraondo et al., 2019). Interleukin-2 (IL-2) and interferon-alpha (IFN-α) were among the first cytokines used in oncology, showing clinical benefit in subsets of patients with metastatic melanoma and RCC by enhancing cytotoxic immune activity. However, their clinical use has been hindered by systemic toxicity, including vascular leak syndrome, neuropsychiatric effects, and multiorgan dysfunction, largely due to their pleiotropic nature (Saxton et al., 2023). To improve safety and efficacy, efforts have focused on engineering cytokines with extended half-lives (e.g., pegylation), tumor-targeted delivery systems, and combinations with other immunotherapies (Aung et al., 2023). Notably, combining cytokines with ICIs or mAbs is being investigated to boost antitumor immunity while minimizing adverse effects (Hansel et al., 2010). Advances in cytokine engineering and biomarker-driven approaches are expected to refine patient selection and therapeutic outcomes. To address the limitations of pleiotropy and systemic toxicity, cytokine mimetics have emerged as a promising strategy. Using computational design, *de novo* mimetics such as Neo-2/15, engineered by David Baker’s lab, selectively engage IL-2 receptor βγ chains, avoiding CD25-mediated toxicities while enhancing effector T cell activity and antitumor efficacy (Silva et al., 2019). These mimetics offer improved pharmacokinetics, reduced off-target inflammation, and greater tumor selectivity. To enhance tumor targeting and minimize systemic exposure, a variety of delivery technologies are under development. Nanoparticle-based carriers, hydrogels, and immunocytokine conjugates offer controlled release, preferential tumor accumulation, and improved pharmacokinetics. For instance, LNP-encapsulated cytokine mRNAs (e.g., IL-7 + IL-21) delivered intratumorally induced strong CD8^+^ T cell responses and durable antitumor immunity in preclinical settings (Hamouda et al., 2024). Reviews highlight how nanocarriers enhance efficacy of cytokines via localized delivery while minimizing systemic toxicity (Wang et al., 2025).

### Oncolytic viruses

OVs represent a novel class of cancer therapeutics capable of selectively infecting and lysing tumor cells while sparing normal tissues (Chen et al., 2023; Rivera-Orellana et al., 2025) (Figure 1D). Their antitumor activity is mediated through direct oncolysis and the induction of systemic immune responses (Ma et al., 2023). Upon infection, OVs induce cellular stress pathways and stimulate the release of type I interferons (IFNs), activating DCs, cytotoxic CD8^+^ T lymphocytes, and NK cells (Kaufman et al., 2015). Tumor lysis releases PAMPs and DAMPs, TAAs, and neoantigens, promoting both innate and adaptive immune activation (Sun et al., 2023). Among OVs, talimogene laherparepvec (T-VEC), a modified herpes simplex virus type 1 (HSV-1) encoding granulocyte-macrophage colony-stimulating factor (GM-CSF), is the most clinically advanced and FDA-approved for metastatic melanoma (Coffin, 2016). Clinical results from the OPTiM Phase III trial demonstrated improved overall and durable response rates compared to conventional therapies (Andtbacka et al., 2015). Other platforms under investigation include HSV-based vectors (e.g., HF10, HSV1716) (Reddy et al., 2024), adenoviruses (e.g., Oncorine, ONYX-015, CG0070) (Fukuhara et al., 2016; Geoerger et al., 2002), reovirus (e.g., Reolysin, targeting Ras-mutated tumors) (Norman and Lee, 2000), and vaccinia virus (e.g., JX-594, also expressing GM-CSF) (Cousin et al., 2022; Lee et al., 2023). These agents exploit tumor-specific vulnerabilities to enhance selectivity and immune engagement. OVs are increasingly evaluated in combination with ICIs, chemotherapy, and radiotherapy to amplify efficacy and overcome immune resistance. Ongoing developments focus on novel viral platforms, such as measles virus, Newcastle disease virus, and Zika virus, and biomarker-guided strategies to personalize therapy and expand clinical utility across diverse cancer types (Zhang and Rabkin, 2021).

### Synthetic cationic helical polypeptides

Synthetic cationic helical polypeptides have emerged as a novel class of immunotherapeutic agents capable of inducing potent antitumor immune responses, particularly through the activation of APCs. These engineered polypeptides represent a promising strategy for the treatment of advanced and metastatic breast cancer by engaging critical innate immune pathways (Lee et al., 2024). Upon exposure, they activate key intracellular sensors including TLR9, cyclic GMP-AMP synthase (cGAS), and the stimulator of interferon genes (STING) adaptor protein. This activation cascade leads to the production of IFNs, which in turn stimulate robust cytotoxic T cell responses directed against tumor cells (Vormehr et al., 2019). The therapeutic efficacy of these polypeptides is rooted in their precisely designed physicochemical properties—electrostatic charge, hydrophobicity, and helical secondary structure. These features enhance systemic activity, immunogenicity, and serum stability (Lee et al., 2024). Mechanistically, synthetic polypeptides induce endoplasmic reticulum (ER) stress in APCs, prompting the release of mitochondrial DNA (mtDNA) into the cytosol. This mtDNA acts as a danger signal that activates innate immune pathways, thereby augmenting inflammatory responses, enhancing antigen presentation, and promoting phagocytosis of tumor cells by APCs (Krysko et al., 2012). Among the different polypeptides investigated, protamine alpha (PTMA), P1, and PS exhibited distinct characteristics in terms of serum stability, hydrophilicity, and pro-inflammatory gene induction. The P1 polypeptide emerged as the most promising candidate due to its optimized helical conformation, achieved through the incorporation of ethylene glycol moieties. This structural refinement endowed P1 with superior serum stability, prolonged circulation time, and enhanced tumor accumulation (Lee et al., 2024). In preclinical models, P1 effectively suppressed tumor growth and promoted the infiltration of IFNγ-producing T cells into the TME. It also improved APC-mediated phagocytosis and supported memory T cell development, critical for achieving durable antitumor immunity. Combination therapy using P1 and anti-PD-1 checkpoint blockade further enhanced antitumor efficacy. This combinatorial approach synergistically activated both TLR9-MyD88 and c-GAS-STING pathways, leading to the phosphorylation and nuclear translocation of interferon regulatory factor 3 (p-IRF3), a central transcription factor for IFN production. In murine models of 4T1 breast cancer brain metastases, this strategy significantly reduced tumor burden and prolonged survival, with 25% of treated mice surviving up to 150 days without evidence of tumor recurrence (Lee et al., 2024). From a clinical perspective, synthetic cationic helical polypeptides offer multiple advantages, including synthetic tunability, selectivity for immune activation without inducing global inflammation, scalable production, and the potential for systemic administration with limited off-target effects (Zheng et al., 2025). However, some limitations remain, such as limited pharmacokinetic and toxicity data in humans, the need for improved tissue targeting in complex tumor environments, and uncertainty regarding long-term immune consequences of sustained innate activation (Svenson et al., 2022). Further optimization of their structure, formulation, and delivery platforms will be critical for successful translation into clinical practice (Weiss et al., 2022). The rational design of synthetic cationic helical polypeptides represents a transformative direction in cancer immunotherapy, particularly for solid tumors. P1’s ability to trigger intracellular DNA sensors via ER stress-induced mtDNA release exemplifies its multifunctional immunostimulatory capacity. By integrating molecular engineering with immunologic insights, these polypeptides offer a versatile and effective platform for inducing systemic and durable antitumor responses.

## Current landscape of combination therapy strategies

Combination immunotherapy has become essential for overcoming resistance mechanisms that limit the durability of ICI monotherapies. Guided by biomarkers and tumor profiling, current strategies aim to enhance efficacy by simultaneously targeting immune suppression, tumor-intrinsic pathways, and the TME (Goswami et al., 2024; Sharma et al., 2023) (Table 1).

**TABLE 1:** Combination immunotherapy strategies.

Combination category	Representative examples	Strengths/mechanistic rationale
ICI + chemotherapy	Nivolumab + platinum doublet chemo (NSCLC, CheckMate 9LA)	Enhances antigen release and T cell priming; converts cold tumors into inflamed tumors
ICI + targeted therapy	Atezolizumab + bevacizumab (HCC, IMbrave150)	Normalizes vasculature and improves T cell infiltration; overcomes resistance to monotherapy
ICI + radiation	Durvalumab + chemoradiation (NSCLC, PACIFIC trial)	Induces immunogenic cell death; enhances antigen presentation and T cell recruitment
ICI + cancer vaccines	mRNA-4157/V940 + pembrolizumab (KEYNOTE-942)	Boosts T cell priming and broadens TAA recognition; enhances immune memory
ICI + oncolytic viruses	T-VEC + ipilimumab (melanoma, NCT01740297)	Enhances local inflammation and T cell trafficking; synergistic antitumor activity
ICI + microbiome modulation	FMT + anti-PD-1 in melanoma	Restores immune tone and ICI sensitivity; improves patient stratification
CAR T + checkpoint blockade	CAR T cells + PD-1 blockade (preclinical and early trials)	Overcomes CAR T exhaustion and immunosuppression in TME
CAR T + cytokines or small molecules	IL-15 superagonists + CAR T (e.g., N-803, preclinical)	Improves CAR T persistence, expansion, and cytotoxicity

### Combination strategies with immune checkpoint inhibitors

The development of combination immunotherapy has become essential to overcoming the resistance mechanisms that limit the efficacy of ICIs as monotherapies. Dual checkpoint blockade, such as the combination of ipilimumab (CTLA-4 inhibitor) and nivolumab (PD-1 inhibitor), has demonstrated superior efficacy and received FDA approval for multiple cancers, including melanoma, NSCLC, RCC, HCC, and microsatellite instability-high (MSI-high) colorectal cancer (Livingstone et al., 2022; Olson et al., 2021; Wolchok et al., 2022; Butterfield and Najjar, 2024). The approval of nivolumab with the LAG-3 inhibitor relatlimab for advanced melanoma underscores the expanding therapeutic potential of targeting additional inhibitory receptors (Tawbi et al., 2022; Cillo et al., 2024). Combinations under clinical evaluation include PD-L1 inhibitors (atezolizumab and dostarlimab) with TIGIT (tiragolumab) or TIM-3 (cobolimab) antagonists, as well as bispecific antibodies (LY3415244) that engage multiple checkpoints (Cho et al., 2022; Rousseau et al., 2023; Hsu et al., 2024; Zhang et al., 2025; Acharya et al., 2020; Sauer et al., 2023; Hellmann et al., 2021). Beyond inhibitory checkpoints, costimulatory agents offer another promising avenue. These agents enhance T cell activation and effector function, especially when used alongside inhibitory ICIs. For instance, pembrolizumab (anti–PD-1) is being evaluated in combination with INBRX-106, an OX40 agonist, for advanced solid tumors (Postel-Vinay et al., 2023). Similarly, combinations of PD-1 inhibitors like pembrolizumab or spartalizumab with TRX518, a GITR agonist, are being tested in solid tumors and lymphomas (Geva et al., 2020). Cytokines are also being integrated into combination regimens to enhance immune activation. IL-12 and IFNs can promote a proinflammatory TME by enhancing DC activation and antigen presentation, while IL-2 supports T cell proliferation and survival. Ongoing studies are evaluating ICIs in combination with IL-12, IL-2, or IFNγ across indications such as melanoma, solid tumors, and lymphomas (Zibelman et al., 2023; Algazi et al., 2020).

### Integration with conventional and targeted therapies

ICIs are increasingly combined with conventional therapies to enhance immune priming and tumor clearance. Chemotherapy induces immunogenic cell death and facilitates antigen release, augmenting the effectiveness of checkpoint blockade. Clinical trials have evaluated combinations such as nivolumab with gemcitabine and cisplatin in esophageal squamous cell carcinoma, NSCLC, and advanced cervical cancer (Doki et al., 2022; Forde et al., 2022; van der Heijden et al., 2023), as well as atezolizumab with carboplatin and etoposide for SCLC (Horn et al., 2018; Galluzzi et al., 2020). Radiotherapy promotes a proinflammatory microenvironment and antigen exposure, with several trials exploring its synergy with ICIs in solid tumors. For instance, durvalumab (anti–PD-L1) and tremelimumab (anti–CTLA-4) have been tested in combination with hypofractionated radiotherapy for NSCLC (Chang et al., 2022; Schoenfeld et al., 2022; Galluzzi et al., 2023), while stereotactic radiotherapy combined with nivolumab and ipilimumab has shown efficacy in Merkel cell carcinoma (Kim et al., 2022). Neoadjuvant and adjuvant strategies that incorporate ICIs are being evaluated in early-stage clinical trials to improve long-term immune surveillance and prevent recurrence. Trials include neoadjuvant regimens such as dostarlimab for dMMR colorectal cancer, nivolumab plus ipilimumab for melanoma, atezolizumab for HER2-positive breast cancer, and pembrolizumab for early-stage NSCLC (Chalabi et al., 2024; Blank et al., 2024; Wakelee et al., 2023; Huober et al., 2022). Adjuvant applications of pembrolizumab and atezolizumab are being studied for NSCLC and RCC (Choueiri et al., 2021b; Felip et al., 2021). In parallel, ICIs are being integrated with targeted therapies, including tyrosine kinase inhibitors (TKIs) and anti-angiogenic agents, which normalize the tumor vasculature and enhance T cell infiltration. Pembrolizumab combined with lenvatinib or axitinib has shown success in RCC and endometrial carcinoma (Makker et al., 2022; Felip et al., 2021; Rini et al., 2019), while nivolumab with cabozantinib is approved for advanced RCC (Choueiri et al., 2021a). Additionally, anti-VEGF therapy combined with ICIs, such as atezolizumab plus bevacizumab, enhances T cell activation and is FDA-approved for HCC (Finn et al., 2020). Targeting oncogenic drivers in melanoma, the combination of atezolizumab with cobimetinib (anti-MEK) and vemurafenib (anti-BRAF) has been approved for BRAF-V600E-mutant melanoma (Ascierto et al., 2023).

### Emerging and innovative combinations

Innovative approaches are rapidly expanding the therapeutic scope of immunotherapy. One major area of progress involves personalized neoantigen vaccines, which aim to elicit tumor-specific T cell responses. These vaccines are being evaluated in combination with ICIs, such as ipilimumab or nivolumab, in melanoma, NSCLC, urothelial carcinoma, and glioblastoma multiforme (Ott et al., 2017; Ott et al., 2020; Awad et al., 2022). Similarly, bispecific T cell engagers (e.g., blinatumomab and PSMA-targeting BiTEs) are designed to redirect T cells toward tumor cells and are under investigation in both solid and hematologic malignancies (Kamat et al., 2021; Deegen et al., 2021). Another promising avenue involves the integration of adoptive cell therapy with ICIs. CAR T cell therapies co-administered with PD-1 or PD-L1 inhibitors are being explored to overcome T cell exhaustion and the immunosuppressive TME (Zhao et al., 2015; Grosser et al., 2019). In parallel, IL-15 superagonists such as N-803 have shown the potential to enhance CAR T cell persistence and antitumor efficacy in both preclinical and early-phase clinical studies (Romee et al., 2018; Lui et al., 2023). Radiotherapy is also being combined with ICIs to potentiate immune responses by promoting antigen exposure and proinflammatory signaling. Hypofractionated and stereotactic radiotherapy regimens have been tested with durvalumab (PD-L1 inhibitor) and tremelimumab (CTLA-4 inhibitor) in NSCLC (Pakkala et al., 2020; Twyman-Saint Victor et al., 2015; Zhang et al., 2022), and in combination with nivolumab and ipilimumab in Merkel cell carcinoma (Kim et al., 2022). These approaches aim to convert immunologically “cold” tumors into “hot” ones that are more susceptible to immune attack. OVs, such as T-VEC, have also demonstrated synergy with ICIs by inducing immunogenic cell death and amplifying systemic immune activation, particularly in melanoma (LaRocca and Warner, 2018; Dong et al., 2023). In parallel, metabolic modulators are being developed to counteract immunosuppressive metabolites like lactate within the tumor microenvironment, thereby restoring T cell function (Zheng et al., 2024). Another Frontier in combination immunotherapy involves modulation of the gut microbiota. Probiotics, prebiotics, and dietary interventions are being tested as adjuncts to ICIs to enhance systemic immune tone and therapeutic response (Lu et al., 2022). Finally, novel multi-checkpoint blockade strategies are emerging. A recent neoadjuvant regimen involving simultaneous inhibition of PD-1, CTLA-4, and LAG-3 demonstrated durable tumor control in glioblastoma, with no recurrence at 17 months. This promising result has prompted the launch of a dedicated clinical trial (GIANT, NCT06816927) to further investigate this triple-combination approach (Long et al., 2025). Together, these innovative combination strategies exemplify the future of precision immuno-oncology, offering the potential to overcome therapeutic resistance, enhance efficacy, and deliver durable benefit across a wide range of tumor types.

## Conclusion and future perspectives

Cancer immunotherapy has revolutionized oncology by mobilizing the immune system to target malignant cells, most notably through ICIs. Despite durable responses in select malignancies, many patients experience primary or acquired resistance due to tumor heterogeneity, immunometabolic rewiring, and the immunosuppressive TME (Wang et al., 2022). These limitations have catalyzed the development of next-generation immunotherapies, including novel inhibitory and co-stimulatory checkpoints, bispecific antibodies (Klein et al., 2024), adoptive cell therapies (Morotti et al., 2021), cancer vaccines (Zaidi et al., 2025), cytokine-based interventions (Berraondo et al., 2019), synthetic immunomodulators (Göpfrich et al., 2024), and microbiome-targeted strategies (Sun et al., 2025), each aiming to reprogram antitumor immunity and expand therapeutic efficacy across both solid and hematologic malignancies (Table 2).

**TABLE 2:** Emerging immunotherapeutic strategies in cancer treatment.

Modality	Mechanism of action	Clinical applications	Representative FDA approvals/landmark trials	Strengths and limitations
Immune checkpoint inhibitors	Block CTLA-4 and PD-1/PD-L1 to restore T cell function	Melanoma, NSCLC, RCC, MSI-H CRC	Ipilimumab (CTLA-4) in melanoma (FDA 2011); Nivolumab + ipilimumab in NSCLC (CheckMate 227)	Durable responses in some tumors; limited efficacy in “cold” tumors; risk of irAEs
Cancer vaccines	Stimulate immune response via tumor-specific antigens delivered by DNA/RNA/peptide or DC-based platforms	HPV/HBV prevention, prostate cancer (Sipuleucel-T), pancreatic cancer (mRNA vaccine)	HPV (Gardasil), HBV (Heplisav-B), Sipuleucel-T (FDA 2010); Moderna mRNA-4157/V940 + pembrolizumab (KEYNOTE-942)	Safe and customizable; limited efficacy in poorly immunogenic tumors; slow onset of action
Bispecific antibodies (BiTEs, DARTs)	Redirect T cells to tumor cells using antibodies that bind both TCR (CD3) and TAAs	ALL, MM, DLBCL, SCLC, uveal melanoma	Blinatumomab (BiTE, FDA 2014; for B-ALL); Teclistamab (MM, FDA 2022); Epcoritamab (DLBCL, FDA 2023)	Efficient T cell redirection; short half-life; risk of CRS; complex dosing schedules
Adoptive cell therapies (ACTs)	Reinfuse TILs or engineered T cells (CAR-T, TCR-T) for direct cytotoxicity	B cell malignancies (approved), solid tumors (in trials)	Tisagenlecleucel (Kymriah, FDA 2017), Abecma (idecabtagene vicleucel, FDA 2021); TILs (lifileucel, FDA 2024; melanoma)	Highly specific and personalized; limited efficacy in solid tumors; manufacturing complexity; CRS and neurotoxicity risks
Cytokine therapies	Activate and expand immune effector cells using IL-2, IFN-α, IL-12, *etc.*	Metastatic melanoma, RCC; under evaluation for broader use	High-dose IL-2 (Proleukin, FDA 1992); Bempegaldesleukin (PEG-IL-2, trials in RCC—CheckMate-9ER)	Boosts immune activation; systemic toxicity and narrow therapeutic window; limited tumor targeting
Oncolytic viruses	Infect and lyse tumor cells while triggering innate/adaptive immune responses	Melanoma (T-VEC approved), trials in various solid tumors	T-VEC (Talimogene laherparepvec, FDA 2015 for melanoma); DNX-2401, Pexa-Vec (solid tumor trials)	Direct tumor lysis and immune activation; delivery challenges; antiviral immunity may limit efficacy
Synthetic immunomodulators	Stimulate innate sensors (e.g., STING, TLR9) and promote APC activation and T cell infiltration	Breast cancer models, potentially other solid tumors	STING agonists (ADU-S100, MK-1454 – NCT03937141); TLR9 agonist CMP-001 (melanoma trial NCT02680184)	Activate innate immunity; promising in combination therapies; limited monotherapy efficacy; potential off-target inflammation
Microbiome modulation	Modulate immune tone via gut flora using prebiotics, probiotics, FMT	Predictive of immunotherapy response and irAE risk; under clinical evaluation	Fecal microbiota transplant (FMT) in PD-1 refractory melanoma; SER-109 (CDI, FDA 2023)	Modulates systemic immunity; predictive for ICI response; interindividual variability and regulatory hurdles

A key priority moving forward is the identification of robust biomarkers to guide patient stratification, predict therapeutic outcomes, and enable real-time monitoring of treatment responses. Clinically relevant biomarkers such as tumor mutational burden, microsatellite instability, immune cell infiltration, TGF-β signaling, prior treatment history, and proliferative capacity offer insights into treatment responsiveness (Usset et al., 2024). The integration of multi-omic data, including genomic, transcriptomic, proteomic, metabolomic, and microbiome-derived signatures, will be critical to the evolution of precision immunotherapy, facilitating the development of adaptive, context-specific therapeutic strategies while minimizing off-target toxicity (Sun et al., 2025).

As immunotherapy regimens become increasingly combinatorial, immune-related adverse events (irAEs) are emerging as a major clinical concern (Martins et al., 2019). These toxicities, often affecting skin, liver, lung, gastrointestinal, and endocrine systems, can escalate with the intensity and complexity of treatment. Current management relies on early detection, corticosteroids, and biologic immunosuppressants (Schneider et al., 2021). However, future directions must include predictive biomarkers for irAE susceptibility, identification of molecular drivers, and implementation of prophylactic measures that do not compromise antitumor efficacy. Importantly, growing evidence links gut microbial composition to irAE development and therapeutic response, positioning microbiome modulation (probiotics, prebiotics, dietary changes, fecal microbiota transplantation) as a promising adjunctive approach (Kang et al., 2024).

Innovations in nanotechnology, synthetic biology, and *in vivo* immune cell engineering are reshaping the immunotherapeutic landscape. Lipid nanoparticles, virus-mimetic vesicles, and programmable gene circuits enable precise targeting, context-sensitive activation, and scalable manufacturing of immune effectors. Concurrently, artificial intelligence and systems immunology are being leveraged to decode dynamic tumor–immune interactions, offering tools to personalize treatment regimens and predict long-term outcomes (Kovács et al., 2023; Araujo-Abad et al., 2024). Chronotherapy, optimizing treatment timing based on circadian biology, is also emerging as a means to synchronize immunotherapeutic interventions with host immune rhythms, potentially improving efficacy and reducing toxicity (Bautista et al., 2025; Pérez-Villa et al., 2023).

Addressing disparities in access, response prediction, and trial inclusion remains essential to ensuring that immunotherapy benefits all patient populations. Current biomarker algorithms are often developed in predominantly Caucasian cohorts, limiting their applicability across ethnic groups. Expanding immunogenomic datasets, increasing representation in clinical trials, and studying racial and sex-based variability in immune responses will be vital to achieving global and equitable outcomes (García-Cárdenas et al., 2024; Guerrero et al., 2018; Chua et al., 2025).

Critical research priorities include determining whether early combination therapy can prevent resistance, optimizing sequencing and scheduling of agents to balance efficacy and safety, and further dissecting host–microbiota–immune interactions to enhance outcomes. In parallel, the development of bioresponsive materials, allogeneic cell therapies, and personalized neoantigen vaccines will likely redefine the scope of immunotherapy in coming years.

In conclusion, the future of cancer immunotherapy lies in a multifaceted, biomarker-guided framework that unites mechanistic understanding with clinical translation. By integrating next-generation immune targets, rational combinations, microbiome modulation, and advanced delivery systems, immunotherapy is poised to evolve from a breakthrough intervention into a cornerstone of curative oncology. Achieving this vision will require not only scientific innovation but also commitment to accessibility, inclusivity, and precision across every stage of cancer care.
